# Abdominal obesity, blood glucose and apolipoprotein B levels are the best predictors of the incidence of hypercholesterolemia (2001–2006) among healthy adults: the ATTICA Study

**DOI:** 10.1186/1476-511X-7-11

**Published:** 2008-03-31

**Authors:** Demosthenes B Panagiotakos, Christos Pitsavos, Yannis Skoumas, Yannis Lentzas, Labros Papadimitriou, Christina Chrysohoou, Christodoulos Stefanadis

**Affiliations:** 1 Department of Dietetics – Nutrition, Harokopio University, Athens, Greece; 2 First Cardiology Clinic, School of Medicine, University of Athens, Greece

## Abstract

**Objective:**

In this work we evaluated the 5-year incidence of hypercholesterolemia, in a sample of cardiovascular disease free adult men and women from Greece. We also evaluated the association of several socio-demographic, dietary and lifestyle habits on the incidence of this disorder.

**Methods:**

1514 men and 1528 women (>18 y) without any clinical evidence of cardiovascular disease, living in Attica area, Greece, were enrolled in the ATTICA study from May 2001 to December 2002. The sampling was random, multi-stage, and included information about various socio-demographic, lifestyle (diet, exercise, smoking etc), biological (lipids, and inflammatory markers), and clinical characteristics of the participants. In 2006, a group of experts performed the 5-year follow-up through telephone calls or personal visits (941 of the 3042 (31%) participants were lost to follow-up). Hypercholesterolemia, among people who had normal blood lipids at initial examination, was defined as fasting total cholesterol levels > 200 mg/dl or use of lipids lowering agents (NCEP ATPIII).

**Results:**

The 5-year incidence of hypercholesterolemia was 23.7% (n = 127) in men and 17.7% (n = 110) in women (p for gender differences < 0.001). Multi-adjusted logistic regression analysis which revealed that increased age (odds ratio = 1.05, p < 0.001), waist circumference (odds ratio = 1.02, p = 0.03), fasting blood glucose (odds ratio = 1.01, p = 0.08) and apolipoprotein B (odds ratio = 1.02, p = 0.001) levels, were the most significant baseline predictors of developing hypercholesterolemia within a 5-year period.

**Conclusion:**

Incidence of hypercholesterolemia was high in both genders, emphasizing the burden of this disorder at population level. Aging, increased waist circumference, fasting blood glucose and apolipoprotein B levels were the most significant baseline predictors of hypercholesterolemia.

## Introduction

Hypercholesterolemia, the presence of high levels of cholesterol in the blood, although not a disease, it is a metabolic condition that can be secondary to many diseases, most notably cardiovascular disease [1-3]. The most common cause of elevated serum cholesterol is eating foods that are rich in saturated fats or contain high levels of cholesterol. Elevated cholesterol also can be caused by an underlying disease that raises blood cholesterol levels such as diabetes mellitus, kidney disease, liver disease, or hypothyroidism. It also can be caused by an inherited disorder in which lipoprotein is not metabolized properly by the body. Obesity, in its various manifestations, which generally results from eating a diet high in fat, also can lead to elevated cholesterol levels in the blood [4,5]. As the serum levels of cholesterol increases so did the risk of having coronary heart disease (CHD). It has been suggested that the risk for CHD is approximately five times higher among persons having cholesterol levels of 300 mg/dl or more compared to those with cholesterol levels below 200 mg/dl [1]. According to the Center for Disease Control and Prevention [3], high cholesterol affects about 20% of adults over the age of 20 in the USA, while the highest prevalence occurs in elderly women. The World Health Organization (WHO) [5] reports that high cholesterol contributes to 56% of cases of CHD worldwide and causes about 4.4 million deaths each year. In most parts of the world, the number of female deaths attributed to high cholesterol is slightly higher than the number of male deaths. However, despite these data, detailed information from various parts of the world regarding the distribution and incidence of abnormal blood lipids is sparse in the literature.

Given the lack of current data regarding the incidence of high blood lipids levels in European and, especially south European, populations, like the Greek, the 5-year incidence of hypercholesterolemia (i.e. high total serum cholesterol levels) and its determinants was investigated, in a random sample of cardiovascular disease free adults from Greece.

## Methods

### Study design

The "ATTICA" epidemiological study [6,7] was carried out in the province of Attica (including 78% urban and 22% rural areas) during 2001–2002. The sampling anticipated enrolling only one participant per household; it was random, multistage and based on the age (5 stages), sex (2 stages) distribution of the Attica region (27 stages, census of 2001). Also, all people living in institutions were excluded from the study. During the aforementioned period, 4056 inhabitants from the above area were selected to enrol into the study; of them, 3042 agreed to participate (75% participation rate), 1514 of the participants were men and 1528 were women. All participants interviewed by trained personnel (cardiologists, general practitioners, dieticians and nurses) who used a standard questionnaire.

### Baseline measurements

The baseline evaluation included information about: socio-demographic characteristics (age, sex, mean annual income and years of school, as proxy of social status), personal and family history of hypertension, hypercholesterolemia and diabetes, dietary and other lifestyle habits, such as smoking status, and physical activity. In particular, the evaluation of the nutritional habits was based on a validated semi-quantitative food-frequency questionnaire [8]. All participants were asked to report the average intake (per week or day) of several food items that they consumed (during the last 12 months). Then, the frequency of consumption was quantified approximately in terms of the number of times a month a food was consumed. Any type of alcohol consumption was measured in wineglasses (100 ml) and quantified by ethanol intake (in g per day). In order to describe overall diet composite scores were used, which are necessary for the evaluation of epidemiological associations. Thus, a special Mediterranean diet score was used (range 0–55) that is based on the rationale of the Mediterranean dietary pyramid [9]. Energy intake was calculated through food-composition tables. Smokers were defined as those who were smoking at least one cigarette per day during the past year or had recently stopped smoking (during a year); the rest of the participants were defined as non-smokers. For the ascertainment of physical activity status the International Physical Activity Questionnaire was used (IPAQ, [10]), as an index of weekly energy expenditure using frequency (times per week), duration (in minutes per time) and intensity of sports or other habits related to physical activity (in expended calories per time). Participants who did not report any physical activities were defined as physically inactive (sedentary lifestyle). Body mass index (BMI) was measured as weight (in kilograms) divided by standing height (in meters squared). Obesity was defined as BMI greater than 29.9 Kg/m^2^. Waist (in cm) and hip (in cm) circumferences were also measured. Arterial blood pressure (3 recordings) was measured at the end of the physical examination with subject in sitting position. All participants were at least 30 minutes at rest. Participants whose average blood pressure levels were greater or equal to 140/90 mmHg or were under antihypertensive medication were classified as having hypertension, as it is commonly done in epidemiological studies. Blood samples were collected from the antecubital vein between 8 to 10 a.m., in a sitting position after 12 hours of fasting and alcohol abstinence. Total serum cholesterol, HDL-cholesterol, and triglycerides were measured using chromatographic enzymic method in a Technicon automatic analyser RA-1000 (Dade Behring, Marburg, Germany). Hypercholesterolemia was defined as total cholesterol levels greater than 200 mg/dl or the use of lipids lowering agents (NCEP ATPIII [11]). HDL cholesterol was determined after precipitation of the Apolipoprotein B containing lipoproteins with dextran-magnesium-chloride. Serum for the measurement of these lipids was harvested immediately after admission. LDL cholesterol calculated using the Friedewald formula: {total cholesterol} – {HDL cholesterol} – 1/5 (triglycerides) (only in people who had triglycerides levels lower than 250 mg/dl). The intra and inter-assay coefficients of variation of cholesterol levels did not exceed 9%, triglycerides 4% and HDL 4%. Diabetes mellitus as a fasting blood sugar > 125 mg/dl or the use of antidiabetic medication. Details about the ATTICA study may be found elsewhere [6].

### Follow-up

During 2006, the ATTICA study's investigators performed the 5-year follow-up. Of the 3042 initially enrolled participants, 1012 men and 1035 women were alive at the time of the follow-up (69% participation rate), while 32 (2.1%) men and 22 (1.4%) women died during the 5-year period. Of the participants that they did not participate in the re-examination (n = 995), 75% were not found because of missing or wrong addresses and telephone numbers, and 25% denied being re-examined. No significant differences were observed between those who were lost to follow-up and the rest of the participants regarding sex (p = 0.99), and baseline age (p = 0.78), education level in years of school (p = 0.67), as well as presence of hypertension (p = 0.12), diabetes (p = 0.27) and hypercholesterolemia (p = 0.12).

The re-examination was based on telephone interviews (80% of the participants) and on face-to-face interviews when the telephone number was not available. The re-examination included information about: (a) vital status (death from any cause or due to cardiovascular disease), or development of CHD (including myocardial infarction, angina pectoris, other identified forms of ischemia -WHO-ICD coding 410–414.9, 427.2, 427.6-, heart failure of different types, and chronic arrhythmias -WHO-ICD coding 400.0–404.9, 427.0–427.5, 427.9-) or development of stroke (WHO-ICD coding 430–438), (b) development of hypertension, hypercholesterolemia, and diabetes, among people who did not have these disorders at baseline, as well as management of these conditions, (c) assessment of body weight and height, and (d) lifestyle habits, including physical activity and smoking status, as well as various food groups intake. For the present analysis people who have been defined as having hypercholesterolemia at baseline examination (n = 1140) were excluded. Thus, taking into account those who were lost to follow-up and those who had high cholesterol levels at baseline examination, data from 1157 participants were analyzed in this work.

### Statistical analysis

Continuous variables are presented as mean values ± standard deviation. Categorical variables are presented as absolute and relative frequencies. Associations between categorical variables were tested using the chi-squared test. Comparisons of mean values of normally distributed continuous variables between those who developed hypercholesterolemia and the rest of the participants were performed using Student's t-test, after controlling for equality of variances. Since the exact time to event (i.e., development hypercholesterolemia) was not available, the relative risks of developing this disorder during the 5-year period, according to the participants' baseline characteristics, were estimated using the odds ratios (OR) and their corresponding 95% confidence intervals (CIs) through stepwise multiple logistic regression analysis. We used 5% as the cut-off for the probability of entering a variable in the model and 10% as the cut-off for the probability of removing a variable from the model. The probabilities were calculated using the Wald test. Interactions between sex and other covariates were tested in all steps, and when they were significant, remained in the final model. Deviance residuals were used to evaluate model's goodness-of-fit and -2loglikelihood of the initial and final model were also calculated. Finally, to further explore which of the baseline measurements was the best predictor of hypercholesterolemia a discriminant analysis was performed using the Area Under the Receiver Operating Characteristic Curve (AUC, higher values of the AUC, better discriminating ability). Moreover, positive and negative predictive values were also calculated for the predictors that remained significant in the multiple logistic regression analysis. All reported *P*-values are based on two-sided tests and compared to a significance level of 5%. SPSS version 14 (Statistical Package for Social Sciences, SPSS Inc, Chicago, IL, U.S.A.) software was used for all the statistical calculations.

## Results

The 5-year incidence of hypercholesterolemia was found 23.7% (n = 127) in men and 17.7% (n = 110) in women (p for gender differences < 0.001). Thus, the annual incidence of hypercholesterolemia is calculated to be 474 new cases per 10,000 men and 354 new cases per 10,000 women participants. A linear trend was observed between incidence of hypercholesterolemia and age – group of the participants (p for trend < 0.001, Table 1). In particular, men who were >65 y at baseline examination experience 5.5-times higher risk of developing hypercholesterolemia compared to those who were under 35 y, while women over 65 y at baseline experience 15.2-times higher risk of developing hypercholesterolemia compared to < 35 y old participants. Furthermore, the men-to-women incidence rate ratio is 1.34; however, a gradual change is observed at the age of 55 y and older, where older women seem to be more prominent to develop hypercholesterolemia compared to men (Table 1). A strong association was also observed between baseline obesity status and incidence rates in both genders (p < 0.001). In particular, new cases of hypercholesterolemia were 16%, 26% and 32% among normal weight, overweight and obese men, as well 12%, 31% and 23% among normal weight, overweight and obese women, respectively. Additionally, incidence rates of hypercholesterolemia were 2-times higher in women who had had hypertension compared to normotensives (i.e. 28.7% vs. 14.8%, p < 0.001), while no differences regarding the incidence of hypercholesterolemia was observed between hypertensive and normotensive men (i.e. 25.9% vs. 22.1%, p = 0.34). Finally, incidence of hypercholesterolemia was almost 3-times higher in diabetic women (i.e. 45.5% vs. 16.7%, p < 0.001), and 2 – times higher among diabetic men compared to those who had normal glucose levels at baseline examination (i.e. 45.9% vs. 22.0%, p = 0.001).

**Table 1:** Five-year incidence of hypercholesterolemia in men and women, by age group.

	Men	Women	
*# People participated in the follow-up*	*536*	*621*	
Age at baseline	5-year incidence of hypercholesterolemia		Men-to-women ratio
<*35 y*	6.90%	4.00%	1.73
*35–45 y*	18.20%	5.00%	3.64
*45–55 y*	29.70%	25.10%	1.18
*55–65 y*	37.80%	57.60%	0.66
> *65 y*	38.10%	60.90%	0.63
Overall	23.70%	17.70%	1.34

Demographic, clinical and behavioural characteristics of the participants by hypercholesterolemia status are presented in Table 2. Although, multiple comparisons are made in the results presented in Table 2, and, consequently the probability of false positives findings (i.e. p-value) increases dramatically, it is noticeable to mention that people who developed hypercholesterolemia within the 5-year period were more likely to be older men, with increased ethanol intake, higher arterial blood pressure levels, increased anthropometric indices, and diabetic, at baseline examination. Of the anthropometric indices waist circumference seems to have the best discriminating ability (AUC = 0.65), followed by BMI (AUC = 0.64) and hip circumference (AUC = 0.60). Moreover, from the baseline blood lipids measurements ROC analysis showed that apolipoprotein B (AUC = 0.75), followed by triglycerides (AUC = 0.727), total cholesterol (AUC = 0.70), LDL-cholesterol (AUC = 0.62), HDL-cholesterol (AUC = 0.55) and apolipoprotein AI (AUC = 0.54) were all significantly associated with the development of hypercholesterolemia.

**Table 2:** Baseline characteristics of the ATTICA study's participants according to the 5-year incidence of hypercholesterolemia.

	Status at 5-year follow – up		
Baseline factors:	Normal total cholesterol (n = 920)	Hypercholesterolemia (n = 237)	P
Age (y)	39 ± 13	50 ± 13	0.001
Male sex, %	44%	54%	0.01
Years of school	13 ± 3	12 ± 4	0.01
Smokers, %	42%	37%	0.21
Physically inactive subjects, %	56%	61%	0.16
Mediterranean diet score (0–55)	27 ± 7	25 ± 6	0.001
Ethanol intake (g/d)	7.0 ± 10	12.2 ± 12	0.001
Systolic blood pressure, mmHg	118 ± 17	125 ± 17	0.001
Diastolic blood pressure, mmHg	77 ± 8	80 ± 10	0.04
Hypertensive subjects, %	23%	33%	0.001
Total cholesterol, mg/dl	163 ± 22	177 ± 19	<0.001
HDL-cholesterol, mg/dl	49 ± 13	47 ± 15	0.06
LDL-cholesterol, mg/dl	98 ± 22	106 ± 21	0.001
Apolipoprotein AI, mg/dl	154 ± 89	149 ± 101	0.02
Apolipoprotein B, mg/dl	88 ± 37	106 ± 23	0.001
Triglycerides, mg/dl	85 ± 47	127 ± 71	0.001
Diabetic subjects, %	3%	11%	<0.001
Fasting glucose, mg/dl	89 ± 19	93 ± 19	0.001
Obese subjects, %	15%	22%	0.008
Body mass index, kg/m^2^	25 ± 5	28 ± 4	0.001
Waist, cm			
*Men*	95 ± 11	100 ± 11	0.001
*Women*	80 ± 12	86 ± 12	0.001
Hip, cm			
*Men*	105 ± 9	106 ± 9	0.10
*Women*	101 ± 11	104 ± 11	0.06

Data are expressed as mean ± SD or relative frequencies.

P-values are not corrected for multiple comparisons.

However, all the aforementioned comparisons are prone to residual confounding; therefore we performed multi-adjusted logistic regression analysis which revealed that increased age (p < 0.001), waist circumference (p = 0.03), fasting glucose (p = 0.08) and apolipoprotein B (p = 0.001) levels, were the most significant baseline predictors of developing hypercholesterolemia within a 5-year period (Table 3). Mediterranean diet score was inversely associated with the development of hypercholesterolemia (*see *initial model), but the relationship became insignificant in the final model because of age and sex differences. Stratified analysis revealed that adherence to the Mediterranean diet was protective only in middle aged (35–55 y) women (relative risk = 0.92, 95% CI 0.87–0.97).

**Table 3:** Results from multiple logistic regression analysis that evaluated socio-demographic, lifestyle, biological, clinical baseline characteristics in relation to 5-year incidence of hypercholesterolemia, in the ATTICA study participants.

*Initial model*	Odds ratio	95% Confidence Interval
Age (per 1 year)	1.05	1.03–1.07
Male vs. females	1.18	0.73–1.87
Waist (per 1 cm)	1.01	0.99–1.03
Years of school (per 1 year)	1.03	0.97–1.09
Fasting blood glucose (per 1 mg/dl)	0.99	0.98–1.01
Total cholesterol (per 1 mg/dl)	1.02	1.00–1.03
Apolipoprotein B (per 1 mg/dl)	1.02	1.00–1.03
Apolipoprotein AI (per 1 mg/dl)	1.00	0.99–1.01
Systolic blood pressure (per 1 mmHg)	1.01	0.99–1.03
Diastolic blood pressure (per 1 mmHg)	0.99	0.97–1.02
Family history of hypercholesterolemia (y/n)	1.18	1.00–1.38
Energy intake (per 100 kcal)	1.02	0.98–1.05
Physically active vs. physically inactive	0.88	0.59–1.32
Smokers vs. non-smokers	1.06	0.79–4.09
Mediterranean diet score (per 1 unit)	0.94	0.90–0.97
Ethanol intake (per 1 g/d)	1.03	1.00–1.07
*Final model*		
Age (per 1 year)	1.05	1.04–1.07
Waist (per 1 cm)	1.02	1.01–1.03
Fasting blood glucose (per 1 mg/dl)	1.01	0.99–1.03
Apolipoprotein B (per 1 mg/dl)	1.02	1.01–1.03
*Final model also adjusted for baseline total serum cholesterol levels*		
Age (per 1 year)	1.04	1.03–1.06
Waist (per 1 cm)	1.01	1.00–1.02
Fasting blood glucose (per 1 mg/dl)	0.99	0.98–1.01
Apolipoprotein B (per 1 mg/dl)	1.005	0.99–1.01

Nevertheless, it is likely that the aforementioned results are explained by the cross-sectional associations of cholesterol and the other variables, like age, sex, blood glucose levels etc. Thus, to further evaluate which factors predict incidence of hypercholesterolemia beyond that of baseline cholesterol, we also included baseline serum cholesterol in the multivariable analyses. Findings suggest that only age, and waist circumference remained significant, after adjusting for baseline cholesterol levels (Table 3), indicating that the predictive ability of the other factors presented in the final model were mainly explained by the baseline differences in cholesterol levels.

Additionally, cut-off point analysis showed that the positive predictive value for age greater than 45 y was 37% (95%CI 35% to 39%), the positive predictive value for waist circumference greater than 95/82 cm (men/women) was 30% (95%CI 28% to 32%), the positive predictive value for fasting glucose greater than 100 mg/dl was 30% (95%CI 28% to 32%) and the positive predictive value for apolipoprotein B greater than 100 mg/dl was 34% (95%CI 31% to 37%). The cut-off points used are the optimal values that discriminate hypercholesterolemic from those who had normal total cholesterol levels at follow-up, as derived from ROC analysis. Moreover, presence of all the aforementioned baseline characteristics (i.e., age > 45 y, waist circumference > 95/82 cm (men/women), fasting glucose >100 mg/dl and apolipoprotein B > 100 mg/dl) 20.2-fold the risk of developing hypercholesterolemia within the 5-year period (95% CI 18.7 to 21.7). In addition, all these factors explained the 22.3% (Negelkerke R-square = 0.223) of the estimated model's variability.

## Discussion

In this work the 5-year incidence rate of hypercholesterolemia was evaluated. In particular, approximately one out of four men and one out of five women that participated in the ATTICA study, developed hypercholesterolemia within this period. The annual incidence rate is about 5% in men and 4% in women, which means that about 900,000 men and women from a total population of 6,5 million people who had had normal cholesterol levels at baseline, developed hypercholesterolemia during the preceding 5 years. Of the baseline factors, increased age, high waist circumference, fasting blood glucose and apolipoprotein B levels, were the most significant determinants of developing hypercholesterolemia.

In the baseline examination (2001–2002), 46% of men and 40% of women had high total cholesterol levels (i.e., >200 mg/dl), and based on these figures it was speculated that about 3,0 million men and women had hypercholesterolemia, in Greece. Based on the present follow up findings, it could be speculated that approximately 180,000 people developed hypercholesterolemia each year, a fact that makes this disorder a very serious problem for the health status of the reference population. The National Health and Nutrition Examination Survey (NHANES) III study [12] reported that 52% of non-Hispanic white men and 49% of women in USA, had total blood cholesterol levels over 200 mg/dl, rates that are similar to those reported by the ATTICA study. Moreover, several studies have shown that a higher percentage of women than men have total blood cholesterol of 200 mg/dl or higher, beginning at age 50 [13,14], which was also observed in the present work since the incidence rate ratio of hypercholesterolemia was higher in women after the age of 55 y (Table 1). Taking into account that the prevalence of hypercholesterolemia in the baseline evaluation of the ATTICA study was about 45%, the Seven Countries Study investigators' [15] reported that in 1980s about 40% of middle-aged Athenian men and women had high total cholesterol levels, while the "Athens Study" investigators [16] also in early 1980s reported similar results, it is now evident that there is a long-term increasing trend of hypercholesterolemia in Greece. This may attribute to the adherence of a more Westernised lifestyle that observed in Greece during the past two decades, which includes consumption of high – fat foods, adoption of sedentary life and increased cigarette smoking [15]. Regarding other populations, in a study conducted in another Mediterranean country, Portugal [17], the investigators reported an incidence of hypercholesterolemia equal to 55.9 cases per 10,000 inhabitants, while there was a higher incidence in men than in women up to the age of 54, but at more advanced ages this relationship was reversed. Although the incidence rates are much lower than those observed in the present study (however, the definition of hypercholesterolemia varied from the one used in this work), a similar trend was observed regarding the men-to-women ratio. Data from the national Nutrition Examination Survey conducted in US from 2001 to 2002, the incidence of combined hypertension and hypercholesterolemia (defined as LDL-cholesterol > 130 mg/dl) was 20% in women and 16% in men, ranging from 1.9% in those aged 20 to 29 years to 56% in those aged greater than 80 years [18]. The latter study underlined the crucial role of hypertension in combination with high blood lipids levels, which also observed in our study, too.

Oster et al., [19] reported that depending on age, gender, and initial BMI level, a sustained 10% weight loss would reduce the expected number of years of life with hypercholesterolemia by 0.3 to 0.8, emphasizing the role of body weight on the development of hypercholesterolemia. In the present work it was observed a 2-times higher incidence of hypercholesterolemia among obese individuals compared to normal weight, in both genders. Moreover, waist circumference was one of the most significant predictors of 5-year incidence of hypercholesterolemia (Table 3); a 10 cm difference in baseline waist circumference levels was associated with 22% (i.e. 1.02^10^) higher risk of hypercholesterolemia within the 5-year period. It is notable that waist circumference was the best predictor among all anthropometric measurements.

Some recent studies have shown that apolipoproteins, and especially apolipoprotein-B/apolipoprotein-AI ratio, predicts cardiovascular risk better than any of the cholesterol indexes [20,21]. It is known that apolipoprotein-B is a primary apolipoprotein of low density lipoprotein and it is responsible for carrying cholesterol to tissues. A recent literature-based meta-analysis showed that compared to participants in the lowest tertile of apolipoprotein-B, those in the highest had 99% increased risk of having CHD [21]. Several investigators have also associated some genetic variations of apolipoprotein-B with the development of hypercholesterolemia and CHD [22-25]. In the present analysis apolipoprotein B was the most significant predictor of hypercholesterolemia among all baseline measurements, including blood lipids. Moreover, 34% of the participants with apolipoprotein B levels greater than 100 mg/dl developed hypercholesterolemia within a 5-year period.

Fasting blood glucose levels were also a significant predictor of hypercholesterolemia. Particularly, 30% of participants who had glucose levels greater than 100 mg/dl at baseline examination developed hypercholesterolemia during the aforementioned period. It is already known that non-insulin-dependent diabetes mellitus is often associated with increased triglyceride, LDL-cholesterol and a reduced HDL-cholesterol levels [26,27]. According to the National Cholesterol education Program, high total cholesterol is present in 70% of adults with diagnosed diabetes and 77% with undiagnosed diabetes in the US population [28]. All these findings demonstrate the relationship between diabetic status and hypercholesterolemia; however, the exact mechanisms that relate high blood glucose levels to the incidence of hypercholesterolemia are not fully understood.

Finally, greater adherence to the Mediterranean diet was inversely associated with the development of hypercholesterolemia (Table 3). It is known that dietary habits usually influence blood lipids levels [29,30]. A traditional dietary pattern that is often consumed in Mediterranean populations has already been related to the reduction of all cause and cardiovascular disease mortality, due to its effect on blood pressure levels, body mass index, platelet aggregation, plasma fibrinogen and other haemostaseological factors [31,32]. In this work it was observed a 6% decrease in the risk of hypercholesterolemia per 1 unit increase in the diet score that assessed adherence of the Mediterranean diet, especially in middle aged women. However, when the analysis was stratified by age and sex, the aforementioned relationship was not significant in men of any age and in older (>55 y) women, because of the masking effect of other covariates included in the statistical models.

### Limitations

The baseline evaluation was performed once, and may be prone to measurement error. Thus, the prevalence of hypercholesterolemia or the levels of total serum cholesterol or other blood lipids, at baseline, may be overestimated. However, our methodology is similar to those of other cross – sectional surveys and follow-up epidemiological studies in Europe and the US, and therefore the results are comparable. The relative risks of developing hypercholesterolemia were estimated by the odds ratios through multiple logistic regression analysis, which may lead to an overestimation of the true effect of the investigated factors. Moreover, multiple significance tests were performed (in Table 2) that may influenced the significance of the findings; however, the results from the multiple logistic regression analysis were the most reliable. Another limitation is that we have not completed the dietary analysis for nutrient components (including electrolytes), so the role of specific nutrients was not evaluated. Finally, telephone calls for the ascertainment of hypercholesterolemia is valid only when accurate medical records exist; therefore, we assessed the 5-year incidence of known hypercholesterolemia and many individuals that developed hypercholesterolemia during the 5-year period, but they did not know it, were misclassified in the re-examination.

## Conclusion

The 5-year incidence of hypercholesterolemia in a population-based sample of cardiovascular disease free adult men and women was evaluated. Roughly one out of four men and one out of five women developed hypercholesterolemia within this period. Age greater than 45 y, waist circumference greater than 95/82 cm, fasting blood glucose greater than 100 mg/dl and apolipoprotein B levels greater than 100 mg/dl, were the most significant determinants of hypercholesterolemia, irrespective of lifestyle habits and other potential confounders. Moreover, adherence to the Mediterranean diet seems to protect from future development of hypercholesterolemia, especially among women. The present work provides information about the incidence rates of hypercholesterolemia and a framework of primary prevention of this metabolic disorder at population level.

## Authors' contributions

DBP designed the study, performed the data analysis and wrote the paper. CP designed the study and critically reviewed the paper. YS, YL, LP, CC participated in the design of the study and data collection. CS critically reviewed the paper.
